# Editorial: Reviews in obstetrics and gynecology 2024

**DOI:** 10.3389/fmed.2025.1661240

**Published:** 2025-07-30

**Authors:** Vittorio Unfer, Artur Wdowiak, Mattia Dominoni, Iwona Bojar

**Affiliations:** ^1^UniCamillus-Saint Camillus International University of Health Sciences, Rome, Italy; ^2^Medical University of Lublin, Lublin, Poland; ^3^Department of Clinical, Surgical, Diagnostic and Paediatric Sciences, University of Pavia, Pavia, Italy; ^4^Department of Obstetrics and Gynecology, Fondazione IRCCS Policlinico San Matteo, Pavia, Italy; ^5^Institute of Rural Health in Lublin, Lublin, Poland

**Keywords:** obstetrics and gynecology, reproductive health, evidence-based medicine, patient-centered care, global health

The field of Obstetrics and Gynecology is rapidly evolving, driven by a growing emphasis on holistic and personalized care. Recent literature suggests looking toward a patient-centered approach, which integrates the social, medical history, and individual background of the specific individual (https://doi.org/10.33178/SMJ.2024.1.18). In this Research Topic, we present reviews, systematic analyses, and case reports that reflect the breadth and complexity of modern practice. The selected works focus on novel obstetric and gynecological insights, ranging from rare diagnostic challenges to large-scale meta-analyses, offering a valuable perspective on current challenges and future directions in the field.

## Case-based insights: enhancing clinical awareness in the management of rare conditions

The case reports highlight the central role of physicians in clinical practice and emphasize that obstetricians and gynecologists must remain vigilant and adaptable when addressing case-to-case variability despite the use of advanced diagnostic tools.

One such report describes a case of a simultaneous intrauterine and intramural gestation in a patient who underwent ART procedures. Heterotopic pregnancy is a rare condition that is poorly described in scientific literature and even more rarely encountered in common practice. In such a case, the ectopic gestation was uncovered thanks to further ultrasound investigations, which allowed prompt intervention and the preservation of pregnancy. While this case enriches the current knowledge on rare obstetric conditions, it stresses the tremendous role of diagnostic imaging in protecting maternal and fetal health in the early phases of gestation.

Similarly, unusual vaginal tuberculosis—a rare form of extrapulmonary infection—posed significant diagnostic challenges as reported by Bicha and colleagues, who illustrated the risk of delayed correct diagnosis. A 35-year-old Ethiopian woman was initially misdiagnosed with pelvic organ prolapse and treated for nearly a year, only to see her condition worsen. The progressive growth of the lesion prompted a more detailed investigation, and specialists established the correct diagnosis. Indeed, the initiation of targeted anti-tuberculosis therapy resulted in marked clinical improvement, underscoring the importance of maintaining diagnostic flexibility, especially in high-prevalence regions such as Ethiopia, where atypical manifestations may occur (Bicha et al.).

When it comes to managing long-term contraceptive complications, a V-shaped copper intrauterine device (IUD), embedded in the uterine wall for over 30 years, was successfully removed using hysteroscopy. The procedure, guided by preoperative imaging and performed with meticulous dissection under direct visualization, allowed clinicians to avoid more invasive surgical interventions. This case highlights the growing potential of minimally invasive techniques in managing long-standing and complex contraceptive device complications (You et al.).

## Psychosocial challenges and preventive innovations in obstetrics and infertility care

Concerning physical health, this Research Topic also deals with the emotional and psychological impact of gynecological and obstetric conditions. Depression among women with infertility is a significant public health concern, especially for those women living in low-resource areas. Alongside economic issues, psychosocial factors such as lack of partner or family support and previous marital difficulties were identified as major contributors to psychological distress. Integrating mental health support into comprehensive infertility care, alongside medical interventions, is an essential factor to consider for improving the overall wellbeing of women who struggle with fertility (Tadesse et al.).

Attention must be paid also during gestation. Preventive strategies are essential in improving maternal health, particularly in reducing the risk of preeclampsia, a major contributor to maternal and fetal complications. Kumsa et al. have shown that calcium supplementation can significantly lower the incidence of this condition. Indeed, calcium plays a key role in vascular function and blood pressure regulation, and its deficiency has been strongly associated with pregnancy-related hypertension (1). The reported findings of this review strongly support calcium supplementation as a cost-effective preventive strategy: it is particularly beneficial during antenatal care in populations with low dietary calcium intake and high risk for preeclampsia.

Further insights into preeclampsia come from research on late-onset cases, a condition that develops after 34 weeks of gestation. Bayilis et al. identified several key risk factors for late-onset preeclampsia, including elevated BMI, advanced maternal age, and chronic hypertension. They suggested that low-dose aspirin may offer a promising preventive approach for high-risk pregnancies, though they emphasized the need for further studies to develop more accurate and clinically applicable predictive models for late-onset preeclampsia.

## Advancements and challenges in reproductive health, oncology, and perinatal care

This Research Topic addresses key advancements and persistent challenges in reproductive health, oncology, and perinatal care, underscoring the need for dedicated attention to women living in poor conditions.

Recent findings suggest that intravaginal misoprostol may offer practical advantages over dinoprostone for labor induction. Comparative studies have shown both drugs to be similarly effective and safe, but misoprostol was associated with a significantly lower need for oxytocin augmentation. Its affordability and ease of administration make it a promising option, particularly in resource-limited settings where access to more complex treatments may be constrained (Lakho et al.).

Moreover, researchers should further investigate the late-gynecological age. In postmenopausal osteoporosis, emerging data support the use of romosozumab as an effective therapeutic option. When added to standard treatment regimens, this monoclonal antibody has been shown to significantly reduce the risk of vertebral and non-vertebral fractures while also improving bone mineral density. Notably, no substantial increase in adverse events has been observed, reinforcing its potential as a valuable addition to current management strategies (Gao et al.).

In cervical cancer research, evidence indicates that women affected by HIV are at higher risk of multiple high-risk HPV infections and developing high-grade squamous intraepithelial lesions. This increased vulnerability highlights the urgent need for improved cervical cancer screening protocols and expanded HPV vaccination efforts, especially in regions with high HPV diversity and limited healthcare access (Cassani et al.).

Finally, a detailed analysis using WHO's International Classification of Diseases for Perinatal Mortality (ICD-PM) revealed strong disparities in global perinatal outcomes. Stillbirth rates were markedly higher in low-income countries, and a considerable proportion of cases remained classified as “unspecified.” These results call for enhanced data collection systems, standardized reporting practices, and targeted interventions to address preventable causes of perinatal mortality worldwide (Kumsa et al.).

## Traditional therapies and methodological challenges

An analysis of clinical trials evaluating traditional Chinese medicine and natural products for endometriosis reveals both promise and limitations. While many studies incorporated key methodological elements such as randomization and blinding, most were limited by small sample sizes and inconsistent design. These findings highlight the need for more rigorous, well-powered trials to accurately assess the potential of complementary therapies in managing chronic gynecological conditions (Zhao et al.).

## Conclusion

Overall, the present Research Topic reflects the dynamic landscape of Obstetrics and Gynecology today, where technological innovation, evidence-based approaches, and global health awareness are increasingly shaping both research and clinical practice. At the same time, they underscore the critical need to integrate psychosocial and systemic perspectives, whether through better mental health support or greater inclusion of complementary medicine.

These insights contribute to a growing dialogue on how best to advance reproductive and women's health through multidisciplinary collaboration, improved methodologies, and patient-centered strategies.
